# Impact of Degraded Aviation Paints on the Aerodynamic Performance of Aircraft Skin

**DOI:** 10.3390/ma18102401

**Published:** 2025-05-21

**Authors:** Wojciech Żyłka, Andrzej Majka, Patrycja Skała, Zygmunt Szczerba, Bogumił Cieniek, Ireneusz Stefaniuk

**Affiliations:** 1 Faculty of Exact and Technical Sciences, Institute of Materials Engineering, University of Rzeszow, Pigonia 1, 35-310 Rzeszow, Poland; bcieniek@ur.edu.pl (B.C.); istefaniuk@ur.edu.pl (I.S.); 2 Department of Aerospace Engineering, Rzeszów University of Technology, al. Powstańców Warszawy 8, 35-959 Rzeszow, Poland; andrzej.majka@prz.edu.pl (A.M.); zygszcze@prz.edu.pl (Z.S.); 3 Faculty of Exact and Technical Sciences, Institute of Physics, University of Rzeszow, Pigonia 1, 35-310 Rzeszow, Poland; pskala@ur.edu.pl

**Keywords:** paint degradation, aerodynamic properties, electron paramagnetic resonance EPR

## Abstract

This study investigates the degradation of aircraft paint and its impact on aerodynamic performance, using the PZL M-20 “Mewa” aircraft as a case study. Paint samples were collected from both damaged and intact areas of the airframe and analyzed using electron paramagnetic resonance (EPR) spectroscopy, scanning electron microscopy (SEM), and aerodynamic testing. One of the major challenges addressed in this work was the non-destructive identification of chemical aging effects in operational paint coatings and their correlation with aerodynamic behavior. The application of EPR spectroscopy in conjunction with real-world aerodynamic testing on naturally degraded surfaces represents an innovative approach that offers both scientific insight and practical guidance for maintenance practices. The results indicate significant deterioration in aerodynamic characteristics—such as increased drag and reduced lift—due to coating damage, particularly around riveted and bolted joints. EPR spectra revealed a notable increase in the density of unpaired electron spins in aged coatings, confirming ongoing oxidative degradation processes. While this study was limited to a single aircraft, the findings highlight the critical importance of regular inspection and maintenance of paint coatings to ensure flight safety and operational efficiency.

## 1. Introduction

According to a recent report by Global Market Insights [1], the global aviation coating market continues to expand, driven by innovations in coating technologies, environmental regulations, and the modernization of aircraft fleets. This growing demand reflects the increasing importance of advanced paint systems not only for corrosion protection and aesthetics but also for aerodynamic efficiency.

The degradation of aircraft paint coatings presents a significant technical and operational challenge in aviation, affecting not only the visual and anticorrosive properties of the surface but also its aerodynamic performance. Paint systems applied to aluminum alloys—commonly used in aircraft structures—typically consist of a conversion layer, primer, and topcoat. Despite this multilayer protection, corrosive processes may still develop beneath the coating, particularly in humid or salt-laden environments, manifesting as blistering, flaking, or surface bubbling [2]. These defects contribute to increased drag and reduced lift and, in extreme cases, may compromise flight control or structural integrity [2,3].

**Figure 1:**
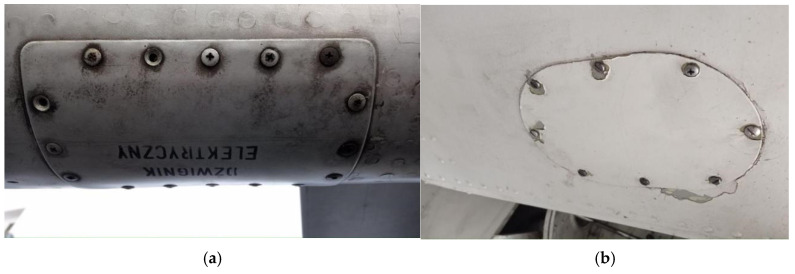
Fragments of aircraft skin: (**a**) Iskra, (**b**) PZL M-20 “Mewa”.

Paint blistering and dark streaks around rivet heads (Figure 1 are common indicators of damage caused by galvanic corrosion, moisture ingress, vibration, mechanical cleaning, and material aging [4,5,6,7,8,9,10,11,12].

In Poland, the sector is largely focused on aircraft maintenance and repair [13], with several facilities—such as Państwowe Zakłady Lotnicze (PZL) Mielec—involved in surface treatment and coating applications. The PZL M-20 “Mewa”, developed for both civilian and military purposes, utilized a variety of paint systems, including epoxy, polyurethane, and acrylic coatings known for their durability and resistance to environmental stressors [14,15,16,17,18,19,20].

Given the increasing complexity of coating systems and operational conditions, advanced diagnostic techniques are necessary for accurate evaluation of coating integrity. One such technique is electron paramagnetic resonance (EPR) spectroscopy, which enables non-destructive identification of chemical degradation processes at the molecular level [21,22,23,24,25,26,27,28,29,30]. EPR has been employed in various research fields, including the analysis of historical pigments [31,32,33,34,35,36,37,38,39], and offers unique advantages for aviation-related applications. It allows for the detection of free radicals, oxidation states, and the breakdown of pigments and binders, providing a deeper understanding of coating aging and deterioration [40,41].

Despite growing awareness of paint degradation and corrosion phenomena, limited research has focused on the aerodynamic consequences of such surface damage. This study aims to address this gap by analyzing naturally aged coatings from a PZL M-20 aircraft and evaluating their effect on aerodynamic performance. The goal is to correlate findings from EPR and SEM analyses with wind tunnel data to better understand the impact of coating degradation and to inform effective maintenance and inspection strategies.

## 2. Materials and Methods

As a sample, aircraft skin from the PZL M-20 “Mewa” (Figure 1b), owned by the Rzeszów University of Technology, was used. The surface of this section exhibits damage to the paint coating resulting from operational use. Samples of paint were taken from the areas around the rivets and the vicinity of screw connections. The analyzed paint samples were compared with the paint coating from an undamaged surface. The collected paint samples were scraped off, cleaned with alcohol, and then dried before further analysis. The analyzed paint samples were compared with the paint coating from an undamaged surface.

**Figure 2:**
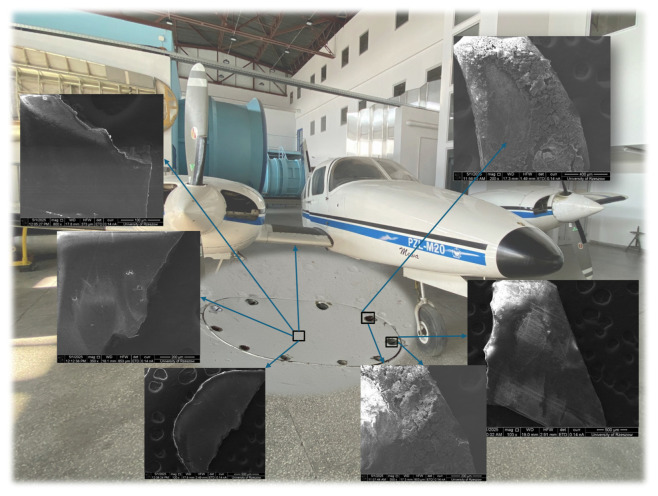
Marked areas on the aircraft skin section: central region—intact paint; rivet zones—degraded coating.

The reference (undamaged) surface was located in the central part of the examined skin section, away from joints and mechanical fasteners. Visual inspection and microscopic analysis confirmed that the coating exhibited a uniform, smooth structure, with no signs of peeling, blistering, discoloration, or surface cracking. This area has been marked in the illustration (Figure 2) to highlight the intact condition of the coating system.

Further analysis was conducted using electron paramagnetic resonance spectroscopy to assess the degradation of the paint coating. The impact of the paint damage on the aerodynamic properties of the components was investigated through aerodynamic testing. Additionally, scanning electron microscopy (SEM) and energy-dispersive X-ray spectroscopy (EDS) were used to analyze the surface morphology and elemental composition of the damaged paint coatings.

Electron paramagnetic resonance (EPR) spectroscopy was used to detect unpaired electrons, which are indicative of degradation-related radicals in the paint matrix. Measurements were carried out using a Bruker ElexSYS E-580 spectrometer (Bruker, Billerica, MA, USA) operating in continuous-wave mode in the X-band (approximately 9.5 GHz). Key spectral parameters were determined, including the resonance field (the magnetic field strength at which microwave absorption occurs), the width of the resonance line (reflecting the uniformity of the chemical environment), and the signal amplitude (related to the number of unpaired electrons formed during degradation processes). Further theoretical background on the principles and interpretation of EPR spectroscopy can be found in references [42,43,44,45,46,47]. Data acquisition and spectral processing were performed using Xepr software (Bruker, https://www.bruker.com/en/applications.html, accessed on 20 March 2025) and the EasySpin toolbox (ver. 6.0.6) for MATLAB (ver. R2024b) [48].

The observed increase in the number of unpaired electron spins in aged coatings indicates the formation of paramagnetic species as a result of chemical degradation processes. These include oxidation reactions leading to the formation of free radicals and the breakdown of molecular bonds in the polymer matrix and pigments.

The broader and shifted EPR lines in degraded samples suggest increased heterogeneity in the local magnetic environment, likely caused by oxidation products and disrupted pigment structures. These findings are consistent with oxidative aging and polymer degradation mechanisms reported in coating degradation studies.

## 3. Results

### 3.1. Scanning Electron Microscope

The presence of elements such as sodium (Na), magnesium (Mg), phosphorus (P), sulfur (S), calcium (Ca), and iron (Fe) on the surface of the aircraft component, particularly in areas where the paint has detached around the holes (Table 1), may indicate processes such as corrosion, contamination, or surface degradation. Sodium and calcium may suggest the presence of salts, potentially originating from seawater or airport de-icing agents, which are known to accelerate corrosion of metals.

Magnesium and iron may derive from corrosion products of metallic sublayers or environmental sources. Their presence may also reflect inadequate surface preparation prior to coating. Residual salts, dust, or industrial pollutants can compromise adhesion and durability of the paint layer.

Phosphorus and sulfur may originate from atmospheric or industrial pollutants or be products of chemical reactions at the metal–paint interface under environmental exposure. Aircraft components are exposed to fluctuating temperatures, humidity, salinity, and industrial emissions, which promote complex degradation processes.

Of particular note is the detection of chlorine (Cl), sodium (Na), and iron (Fe) in degraded samples. Chlorine and sodium are indicative of salt-laden environments, contributing directly to paint layer breakdown. Iron is strongly associated with corrosion by-products and is commonly found in rusted or oxidized areas beneath the damaged coating. These elements were significantly more concentrated in degraded zones than in undamaged areas, confirming the presence of active corrosion and contamination.

In contrast, EDS spectra from undamaged regions showed a markedly lower content of these corrosive or contaminant elements, demonstrating the effectiveness of intact coatings in preventing environmental ingress and preserving material integrity.

These findings support the conclusion that chemical degradation in damaged areas is accelerated by environmental factors and is traceable via SEM/EDS analysis.

To evaluate the surface, internal structure, changes and deformations, as well as the chemical composition of the paint coating in areas exhibiting blistering, filiform corrosion, and visible paint flaking around the rivet holes of the PZL M-20 “Mewa” aircraft, comparisons were made with undamaged areas and paint losses. A scanning electron microscope FEI Quanta 3D 200i (FEI, Hillsboro, OR, USA) with an energy dispersive spectroscopy chemical analyzer was used under high vacuum conditions. A 10 kV accelerating voltage was employed for the chemical composition analysis.

**Figure 3:**
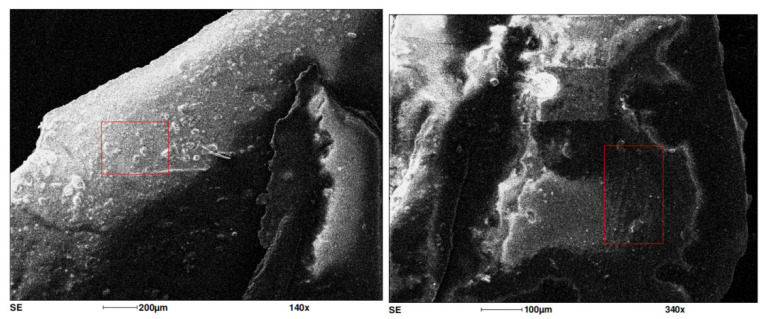
SEM image of the paint sample taken from the vicinity of the rivet hole. The red square marks the area analyzed for chemical composition using EDS.

**Figure 4:**
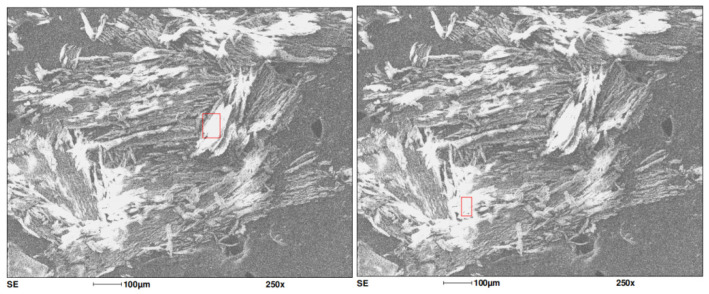
SEM image of the paint sample taken from the undamaged section of the PZL M-20 aircraft skin. The red square marks the area analyzed for chemical composition using EDS.

Microscopic images of the materials obtained using the SE (secondary electrons) detector are shown below in Figure 3 and Figure 4.

SEM images shows differences between the paint samples taken from areas with damaged paint coatings around the mounting holes and the paint samples taken from clean, undamaged surfaces.

### 3.2. Analysis of Detected Elements in Paint Samples

The presence of elements such as sodium, magnesium, phosphorus, sulfur, calcium, and iron on the surface of the aircraft component, particularly in areas where the paint has detached around the holes (Table 1), may indicate issues such as corrosion, contamination, etc. Sodium and calcium may suggest the presence of salts, which often originate from seawater or de-icers used at airports. De-icers are substances or devices used to remove ice and snow from aircraft. Salt is a primary factor that accelerates the corrosion of metals.

- Magnesium and iron: These may derive from corrosion products of other metal components or from environmental contaminants. The presence of these elements may suggest that the surface was not adequately prepared prior to painting. Residues of salts, dust, or other contaminants may have remained on the surface, potentially leading to adhesion issues with the paint.
- Degradation of protective coating: the paint, as a protective coating, may degrade over time due to atmospheric, chemical, or mechanical factors, which can lead to exposure to corrosion.
- Chemical reactions: phosphorus and sulfur may originate from industrial pollutants or be products of chemical reactions occurring on the metal surface in the presence of paint and the external environment.

Environmental impact: Aircraft components are exposed to extreme weather conditions such as temperature fluctuations, humidity, salinity, and industrial pollutants, which can introduce various elements onto the surface. The presence of these elements indicates the need for a more thorough investigation into the preparation, painting, and maintenance processes of aircraft components. Additional testing may also be necessary to identify the sources of these contaminants and implement appropriate preventive measures, such as improved sealing, the use of corrosion inhibitors, or enhanced maintenance procedures.

The presence of chlorine (Cl) and sodium (Na) suggests exposure to salt-laden environments, which accelerates coating degradation. Iron (Fe) is commonly associated with corrosion products that form at or beneath damaged coating layers. These elements were significantly more concentrated in degraded areas, confirming the presence of active corrosion and contamination processes.

In zones affected by corrosion, signs of mechanical degradation may also be observed, such as microcracking, tribological wear, or delamination of the protective layer. These phenomena promote the localized penetration of aggressive agents and initiate further corrosion. The reduced titanium content is attributed to coating loss resulting from mechanical degradation, induced by factors such as microcracking under mechanical loading, residual or operational stresses, and tribological abrasion.

By contrast, EDS spectra obtained from undamaged regions revealed significantly lower concentrations of these elements, confirming the protective effectiveness of intact coatings in preventing environmental ingress.

### 3.3. EPR Measurements

**Figure 5:**
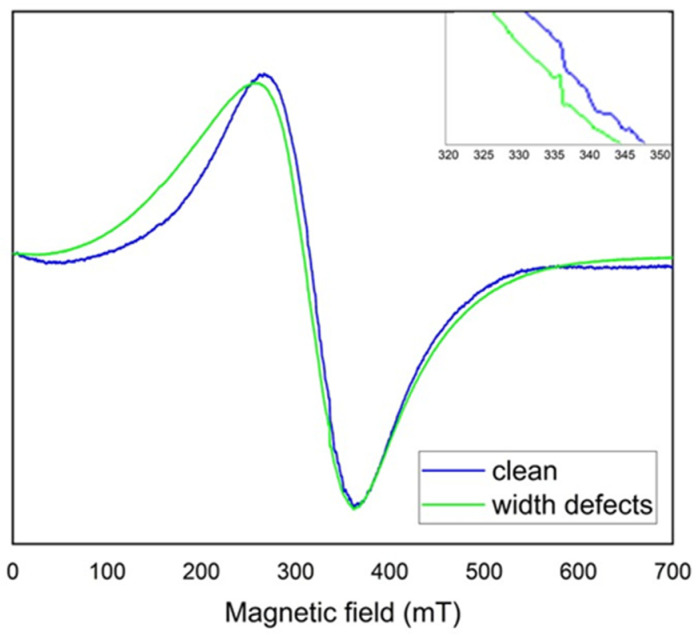
EPR spectrum: —paint sampled near the openings (green line), —paint sampled from an undamaged surface (blue line) + inset view.

To identify the source of contamination, EPR tests were conducted on paint samples taken from defect areas around mounting holes, as well as on samples collected from clean, undamaged sections of the PZL M-20 “Mewa” aircraft skin. Differences can be observed between a paint sample taken from an area of degradation and a clean sample taken from an undamaged part of the aircraft surface. Figure 5 shows the EPR spectra of paints samples.

**Figure 6:**
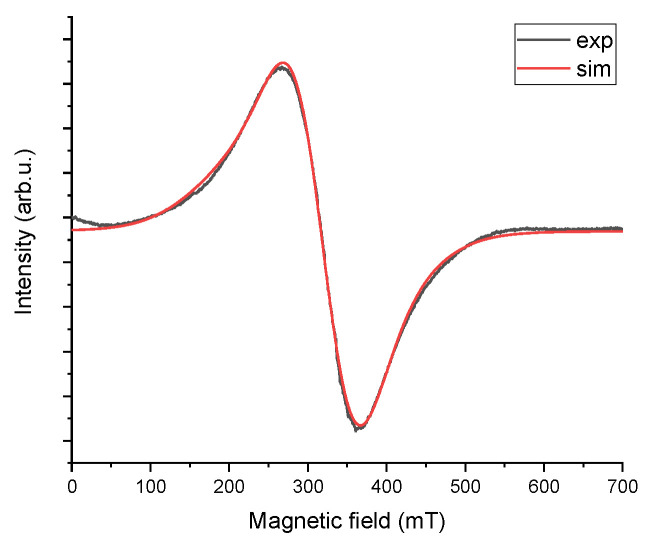
EMR spectrum, clean sample + simulated.

**Figure 7:**
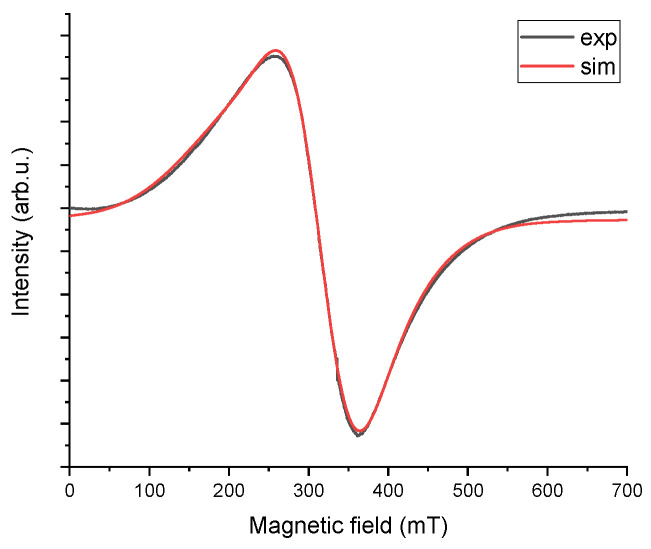
EMR spectrum, sample from area near openings + simulated.

Two lines can be observed—a primary, broad line around 300 mT, with a peak-to-peak width of approximately 100 mT, and a very small one at an effective g factor around 2, located near 350 mT (see inset of Figure 5). Additionally, a broadening of the green line can be seen, along with a slight shift towards a lower field. The differences in the shape of the two lines indicate paint wear and contamination causing degradation of the coating near the mounting holes of the PZL M-20 “Mewa” aircraft’s surface element. The difference in the line shape reveals altered properties of the paint sample marked as defects in Figure 3 compared to the pristine, undamaged surface. A comparison was made between the actual EPR spectra measurements and simulated spectra generated in the Easyspin program within the Matlab package. The EPR spectrum shows two lines (a narrow one originating from carbon) and one broad line, which consists of at least two components. The broad line was analyzed, and spectroscopic parameters were adjusted to fit the two components. The fit demonstrates a high correlation between the experimental spectrum and the simulated one, as shown in Figure 6 for the pristine sample and Figure 7 for the sample taken from near the rivet hole.

The first component of the spectrum (at a lower *g* value and higher B_rez_) is a Voigtian line (Gaussian + Lorentzian). The second component of the spectrum (higher g value lower magnetic field) is a Gaussian line.

For both the experimental and simulated spectra, line parameters were calculated, including intensity, peak-to-peak width, effective g, and resonance field. In Figure 6 and Figure 7, the “sim” line is the sum of the two components. The calculated EPR line parameters are shown in Table 2. Spin calculation procedure were also performed. Because EPR signal is proportional to the number of spins, the number of spins is proportional to the area under an absorption peak. This allows us to determinate of the number of unpaired spins in the samples from the acquired EPR spectrum without the additional steps of preparing and measuring a standard [47]. The value of the number of spins is shown in Table 2 in units of spin per gram. It is readily apparent that the number of spins per gram in the paint sample with defects is one order of magnitude higher.

Analyzing the obtained values, it can be observed that the EPR spectrum of the paint with defects has broadened (H_pp_) along with a slight shift towards a lower field. This is clearly visible in Figure 7. The main contributor to this broadening is the second component (Gaussian line 1.2), which can be seen in Table 2. This component undergoes broadening and a shift towards a lower field. Further analysis should enable the determination of paint wear and air contamination that contributed to this wear. To this end, the following EPR studies should be conducted in the subsequent analysis: calculation of the total number of spins, determination of the spin Hamiltonian of the observed lines.

The observed increase in spin density in degraded samples suggests the formation of paramagnetic centers caused by chemical degradation of the coating. This process is associated with oxidative reactions and the breaking of chemical bonds within the polymer matrix or pigment structures. The presence of unpaired electrons detected by EPR reflects the accumulation of free radicals and metal–organic complexes formed during environmental exposure. These findings confirm that oxidative aging and molecular fragmentation are key mechanisms contributing to the deterioration of the coating’s protective function.

In future work, a more detailed investigation is planned to develop a predictive model of coating aging. This will involve constructing a degradation map of the paint based on changes in Orbach relaxation parameters derived from temperature-dependent EPR measurements. Such an approach will allow for the identification of early-stage chemical degradation processes and enable more accurate assessment of coating durability.

### 3.4. Comparison of Aerodynamic Characteristics of Selected Elements, Models Without and with Paint Coating Damage

**Figure 8:**
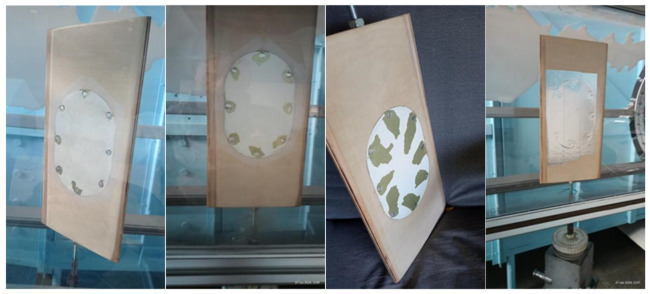
Integrated element from the aircraft “f”, “g”, “i”, “h” into the plane of the profile created in the TA500 wind tunnel.

To determine the impact of paint porosity on the lift surface, a method of investigation was adopted concerning the profile characteristics, and attempts were made to observe the effect of irregularities on the basic characteristics in a qualitative sense. Therefore, an element from the aircraft was integrated into the plane of the profile (Figure 8).

To ensure compatibility with the wind tunnel setup, a physical airfoil model was precisely crafted from wood to match the original wing section of the PZL M-20 aircraft. The tested skin fragment was inserted into the profile flush with the contour to prevent flow disturbance caused by the mounting. This setup allowed for repeatable aerodynamic measurements while preserving the actual degraded surface condition.

The prepared airfoil was installed on an aerodynamic balance using a positioning head that set the angle of attack (Figure 8). In this configuration, the airfoil characteristics Cz and Cx were measured as a function of the angle of attack. Each measurement was repeated four times for every case of paint loss. The aim of the study was to qualitatively assess the impact on profile parameters Cz and Cx, as well as to measure the vibration spectrum generated by vortices induced by changes in surface roughness.

**Figure 9:**
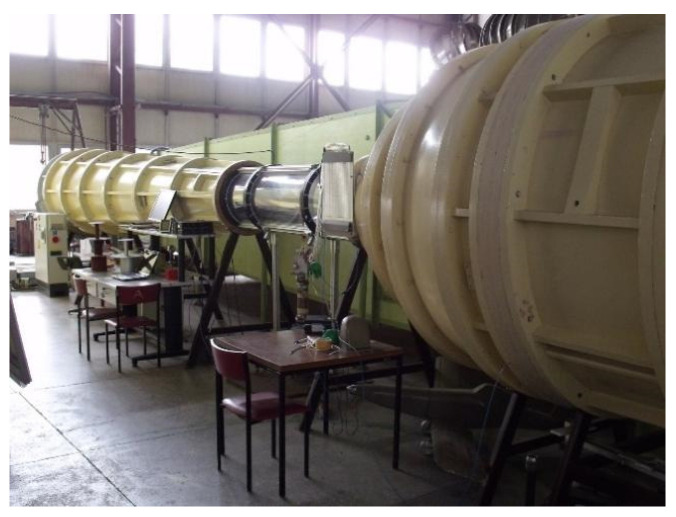
TA500 wind tunnel.

The tests were conducted in the TA500 wind tunnel (Figure 9) with a diameter of 500 mm, operating in both open and closed measurement space configurations, utilizing a columnar aerodynamic balance and the Daqbook 2001 (IOTECH, Norton, MA, USA) measurement system with DasyLab software 2020.

The aerodynamic balance used was a two-component external strain gauge type, measuring the force components Px (aerodynamic drag) and Pz (lift). The measurement system consisted of a Daqbook 2001, configured with strain gauge measurement cards and analog inputs for dynamic pressure measurement. The DasyLab 2020 software platform was used to calculate force values, Cz and Cx coefficients, and flow velocity, as well as to perform statistical RMS analysis and determine the vibration spectrum using the FFT library. Simultaneously, all parameters were monitored on-screen and recorded in real time.

**Figure 10:**
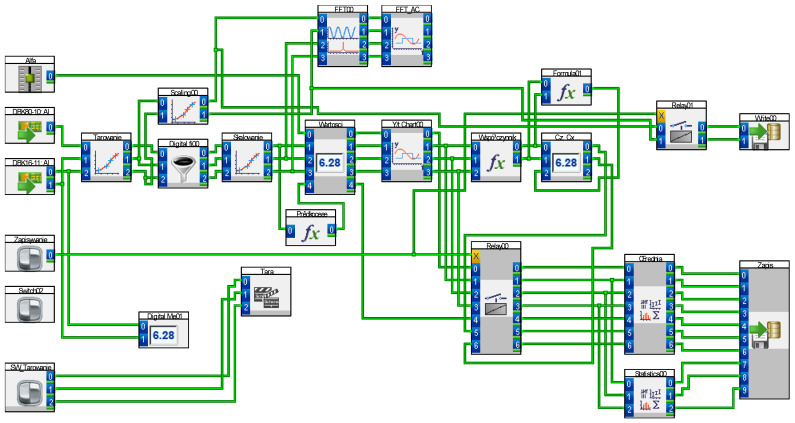
DasyLab measurement diagram for the implementation of tests in the wind tunnel.

The research process was modeled in the DasyLab environment, with the implementation diagram presented in Figure 10.

- Test conditions:
- The determined air density is ρ = 1.16 kg/m^3^;
- The Reynolds number is Re = 346,000 with respect to the model’s chord.
- The study was conducted for four cases:
- h-profile without defects;
- f-profile with the first level of paint loss;
- g-second level of paint surface loss;
- i-third level of paint surface loss.

**Figure 11:**
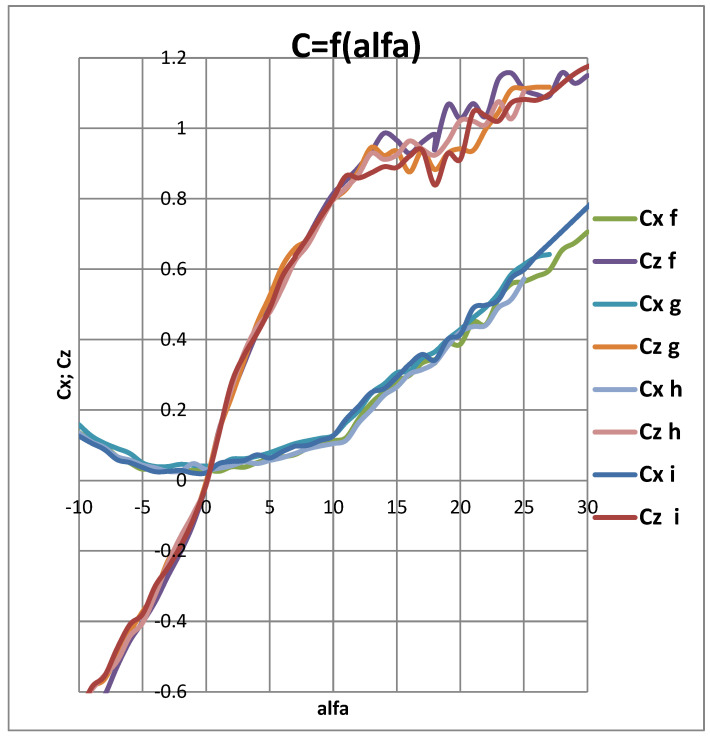
Graph showing the relationship between the aerodynamic drag coefficient (Cx) and the lift coefficient (Cz) as a function of the angle of attack: h-profile without defects; f-profile with the first level of paint loss; g-second level of paint surface loss; i-third level of paint surface loss.

The graph showing the relationship between the aerodynamic drag coefficient (Cx) and the lift coefficient (Cz) as a function of the angle of attack is presented in Figure 11:

- h-profile without defects;
- f-profile with the first level of paint loss;
- g-second level of paint surface loss;
- i-third level of paint surface loss.

In the presented graphs in Figure 11, obtained from tests conducted for four different cases of coating damage, it can be observed that the drag coefficient Cx for the smooth coating and the first stage of damage is significantly lower than in cases “G” and “I.” However, for the lift component Cz, the critical angle shifts to the left by 2–3 degrees, causing earlier flow separation and a reduction in flight performance.

**Figure 12:**
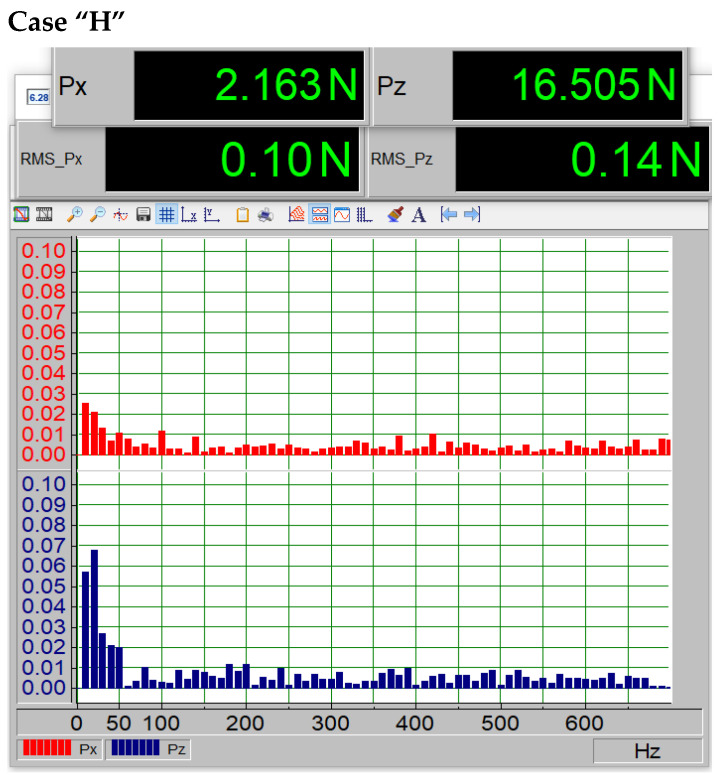
Vibration spectrum of the force components X and Y for the model without paint loss at an angle of attack of 11 degrees.

**Figure 13:**
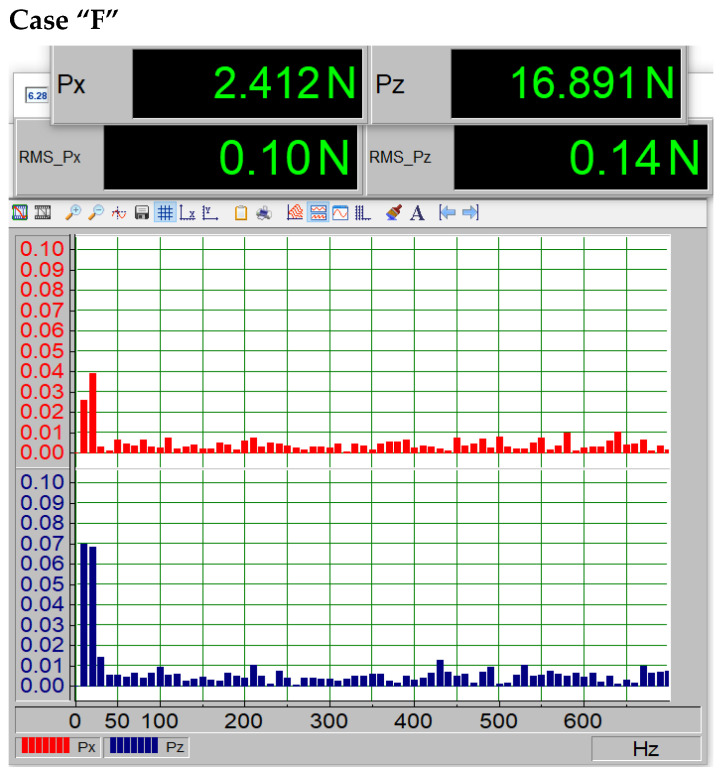
Spectrum and force magnitudes Px and Pz for the first case of paint coating loss (case F).

**Figure 14:**
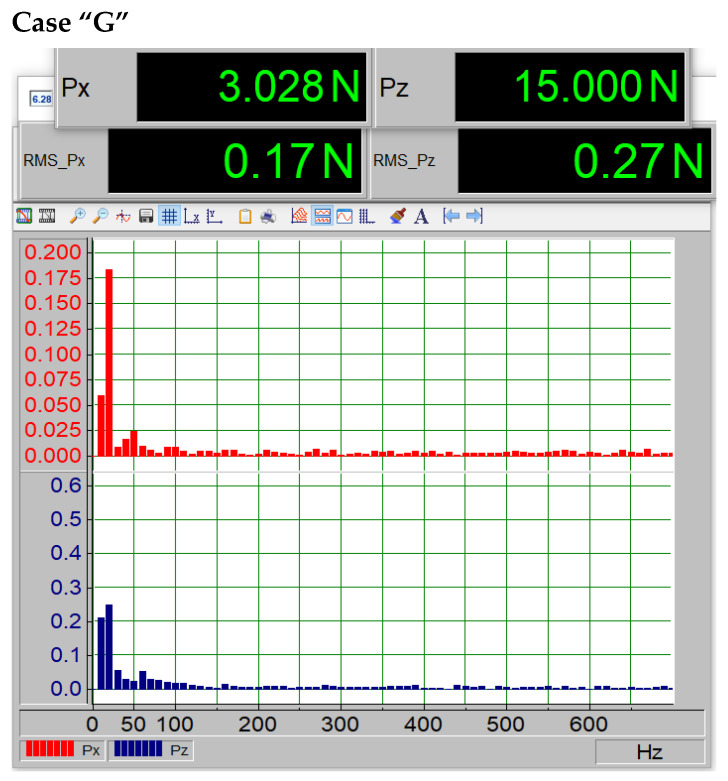
Force magnitudes Px and Pz and vibration spectrum for the second case of paint coating loss (“G”).

**Figure 15:**
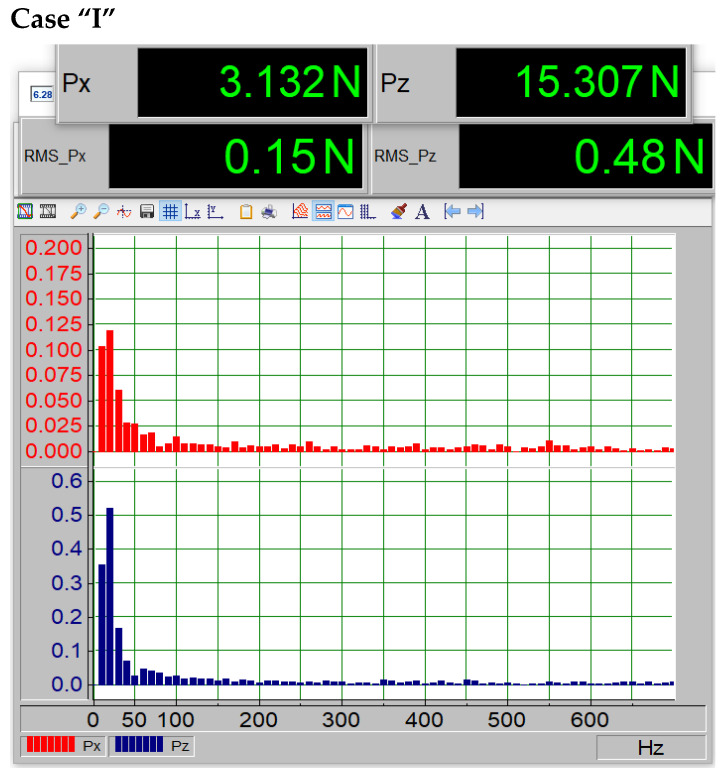
Force magnitudes Px and Pz and vibration spectrum for the third case of paint coating loss (“I”).

In addition to examining the profile characteristics presented in Figure 11, a spectral analysis was conducted using the Fast Fourier Transform (FFT) to determine the spectrum and amplitude of the dynamic components generated on the profile. It should be noted that the porosity was present on the underside of the profile. Figure 11, Figure 12, Figure 13, Figure 14 and Figure 15 show the average force values for the Px and Pz components for the same angle of attack, the root mean square (RMS) values of vibrations, and the spectrum obtained from the FFT analysis of the Px and Pz signals for the same angle of attack.

Table 3 clearly illustrates the relationship between the degree of surface degradation and changes in aerodynamic parameters—as the damage increases, the lift coefficient (Cz) decreases, the drag coefficient (Cx) increases, and the critical angle of attack shifts toward lower values.

Figure 12 presents case “H”, meaning no paint loss, along with the standard deviations (RMS), energy values, and spectral peak amplitudes. The amplitudes of the peaks and RMS values are at a very low level. Based on the nature of the signal spectrum, it can be concluded that the vibrations of the model, caused by air vortices on its surface, have a stochastic character. The dominant harmonics are below 60 Hz.

However, Figure 13 presents case “F”, which corresponds to the first level of paint coating loss. The RMS values and peak amplitudes do not show a noticeable change; the only observable difference with this method is slight variations in the first harmonic frequencies.

In the third case of the study, “G”, which corresponds to the second level of paint coating loss (Figure 14), an increase in RMS values for the Pz component can be observed, with a twofold increase, which is also clearly visible in the FFT spectrum. A noticeable increase in the amplitude of the initial harmonic peaks is evident.

The fourth case of the study, indexed as “I” and shown in Figure 15, clearly demonstrates a fourfold increase in the RMS value of the Pz component. FFT spectrum analysis reveals that the Pz component is nearly 10 times higher than in the first case.

The average forces Px visible on the upper displays in Figure 12, Figure 13, Figure 14 and Figure 15 increase (i.e., drag forces) as the porosity of the paint coating loss increases, while the lift component Pz decreases. This phenomenon is also noticeable in Figure 11. The presented qualitative research results leave no doubt that the presence of paint coating loss on the underside of the wing causes an increase in vibrations, likely due to increased turbulence in the boundary layer, as well as a shift in the critical angle. Such a change in aerodynamic parameters is not without significance for the safety of the device on which it occurs, whether it is an aircraft or a wind turbine. Undoubtedly, this can also affect the efficiency of the device, leading to increased fuel consumption in the case of an aircraft or a car.

The data summarized in Table 4 indicate a clear correlation between surface degradation and vibration characteristics. As the extent of coating damage increases, the root mean square (RMS) value of the vibration signal rises, reflecting higher mechanical instability. Additionally, the dominant frequency shifts toward higher values, which may suggest changes in flow behavior and the onset of turbulent phenomena near the surface. These findings confirm that degradation of the paint layer not only alters aerodynamic performance but also affects dynamic stability.

## 4. Conclusions

We confirmed that damage to paint coatings, particularly around riveted and bolted joints, has a significant impact on the aerodynamic characteristics of aircraft components for the studied geometry and Reynolds number. Blistering has a notably negative effect on aerodynamics and the durability of paint coatings in aircraft components. It leads to an increase in aerodynamic drag, a shift in the critical angle, and a decrease in the maximum lift coefficient, all of which affect the flight characteristics of the aircraft. Moreover, blistering contributes to accelerated corrosion processes and reduces paint adhesion, emphasizing the necessity for careful monitoring and maintenance of paint coatings to ensure the safety and longevity of aircraft. The presence of chemical contaminants accelerates corrosion and degradation of coatings, highlighting the need for thorough surface preparation prior to painting and regular maintenance. These results are crucial for ensuring the longevity and safety of aircraft.

The presented wind tunnel studies clearly indicate that changes in surface quality caused by the loss of paint coating affect the qualitative aerodynamic parameters of the airfoil. As the degraded surface area increases, aerodynamic drag—represented by the dimensionless coefficient Cx—also increases. The lift force, represented by Cz, decreases, and the critical angle of attack is reduced, preventing the achievement of higher Cz values. The vibration levels of the airfoil caused by generated vortices also increase, as evidenced by the RMS indicator, especially in the region of critical angles. All these parameters lead to greater overall losses, which in turn result in higher fuel consumption—for example, in aircraft—thus increasing the emission of harmful oxides into the atmosphere. These findings underscore the importance of maintaining paint coatings in good condition to minimize negative aerodynamic effects, as well as the need for monitoring and maintaining aircraft surfaces to ensure optimal performance and safety of the aircraft.

This study has several limitations that should be acknowledged. All analyses and measurements were conducted using one aircraft type (PZL M-20), and only a specific structural component was examined. Therefore, the results may not be directly generalizable to other aircraft configurations or paint systems. Additionally, degradation processes in different operational environments (e.g., marine, desert, or industrial atmospheres) may exhibit distinct characteristics that were not captured in this work.

Future research should expand the scope of analysis to include various aircraft types, coating formulations, and controlled aging experiments. Further studies could also incorporate time-lapse monitoring of degradation, temperature-dependent EPR analysis, or the development of predictive models for coating lifespan based on environmental exposure and chemical signal changes.

## Figures and Tables

**Table 1:** Energy dispersive X-ray spectroscopy quantitative results.

Element	Wt%	At%	Wt%	At%
	From the Undamaged Section		From the Vicinity of the Rivet Hole	
CK	40.77	56.07	57.65	72.37
OK	33.35	34.44	36.40	33.98
AlK	1.04	0.64	1.63	0.91
SiK	1.27	0.76	4.52	2.43
TiK	25.19	8.81	8.17	2.57
NaK	-	-	2.02	1.32
MgK	-	-	1.22	0.76
PK	-	-	1	0.49
SK	-	-	1.1	0.52
ClK	-	-	0.49	0.21
CdL	-	-	0.96	0.13
CaK	-	-	0.95	0.36
FeK	-	-	3.27	0.88
NiL	-	-	0.46	0.12

**Table 2:** EPR line parameters.

Parameter	Clean	Defects
g_eff_ (1)	2.097	2.123
H_pp_ (1,1)	75.744	66.208
H_pp_ (1,2)	22.984	43.137
g_eff_ (2)	2.239	2.338
H_pp_ (2)	160.668	174.184
Number of spins (spin/g)	2.28 × 10^21^	2.71 × 10^22^

**Table 3:** Summary of aerodynamic parameters for different surface conditions.

Surface Type	Lift Coefficient (Cz)	Drag Coefficient (Cx) α = 5	Critical Angle of Attack (°)
H (smooth)	0.98	0.048	14.2
F (minor damage)	0.94	0.062	13.1
G (moderate damage)	0.92	0.068	11.6
I (severe damage)	0.85	0.079	10.4

**Table 4:** Summary of FFT signal analysis.

Surface Type	RMS_Pz	Amplitude of the Dominant Frequency
H (smooth)	0.14	0.07
F (minor damage)	0.14	0.07
G (moderate)	0.27	0.25
I (severe damage)	0.48	0.52

## Data Availability

The original contributions presented in this study are included in the article. Further inquiries can be directed to the corresponding author.
